# Post Graduate Students’ Experiences with Research Ethics: A South African Perspective

**DOI:** 10.1177/15562646231188004

**Published:** 2023-07-16

**Authors:** Zamandlovu Makola, Nonceba Ntoyanto-Tyatyantsi

**Affiliations:** 1 University of South Africa, Pretoria, South Africa

**Keywords:** postgraduate students, research ethics, student experiences, research ethics principles, research ethics committee

## Abstract

The strict adherence to ethical principles (namely respect for persons, beneficence/non-maleficence and justice) when conducting research involving human participants is the bedrock of research. There has been little research on students’ experiences with the research ethics process and how these students incorporate ethical standards into their research work, despite previous research looking into teaching methodologies, curricula, and the educational environment for postgraduate students. The purpose of the study was to investigate postgraduate students’ experiences with research ethics during their research project. A sample of 11 participants was used. Through online interviews, this qualitative study, underpinned by the interpretivist paradigm, collected data from postgraduate students from different disciplines and universities in South Africa between June and August 2021. The findings showed different perspectives on the training received in research ethics, and on support and guidance received from supervisors, and the application of research ethics principles in their research projects. Most participants indicated gaps in the teaching and support they received and had not read their university research ethics policies. By focusing on students’ experiences in a developing country and different disciplines, the study contributes to the body of knowledge on postgraduate student experiences. Furthermore, the findings suggest that there is need for more research ethics training amongst postgraduate students in South Africa.

## Introduction

Fundamental research ethics principles such as respect for persons, beneficence and justice must be taken into consideration when conducting human studies (National Commission for the Protection of Human Subjects of Biomedical and Behavioral Research, 1979). Many postgraduate students include human participants in their research projects. Thus, students must have a good understanding of research ethics principles to apply them to their individual studies. Students often have a close encounter with research ethics when they apply for their ethical clearance from their universities (DePauw, 2009). Therefore, they need to be provided with ethics training through online modules, courses, or workshops to help them conduct responsible research (Petillion et al., 2017; Swedan et al., 2020). There has been a growing interest in research ethics globally especially in the context of studies involving human participants (Barrow et al., 2023; Burgess et al., 2023; Gefenas et al., 2022). For example, De Costa et al. (2021) explained that research “should not only be a purely intellectual exercise; rather, we need to consider whom we conduct research on, the purpose of our research, the manner in which it is conducted, and the ways in which we disseminate our findings” (p. 60). There are very few studies which have been conducted in Africa and South Africa specifically, on postgraduate students’ knowledge and experiences of conducting responsible research (Maitin-Casalis, 2010; Petillion et al., 2017; Osungbade et al., 2014). Studies conducted outside of the African and South African context cannot be assumed to be applicable to South Africa. Therefore, the purpose of this study was to explore South African postgraduate students’ experiences with research ethics. The study sought to address the following central research question: what are South African postgraduate students’ knowledge and experiences with research ethics principles during their research project?

In the South African context, the research ethics regulatory frameworks have been driven by health and biomedical disciplines (Department of Health, 2015). The Department of Health provides guidelines on the role of Research Ethics Committees (REC); REC membership composition; operational procedures; and standard operating procedures for RECs (Department of Health, 2015). Universities responsible for conducting research have instituted research ethics committees at institutional, faculty/college and departmental levels (Davies, 2020). These RECs are responsible for ensuring sound research ethics governance. Different universities have different processes and procedures for applying for research ethics approval.

### Research Ethics Context in South Africa

The National Health Act (Act 61 of 2003 governs the ethics of scientific research involving human participants in South Africa). The National Health Research Ethics Council (NHREC) was established in 2006 under Section 72 as the regulatory body to:

- To provide oversight of the conduct and procedures of South African human RECs.
- Establish and guide the norms and regulations for research involving human participants (and animals).
- Act as a judicial and disciplinary body to handle grievances and research misconduct.

The Department of Health (2015) *Ethics in Health Research Guidelines* provides the ethical principles applicable to research involving living human participants and animals. Under these guidelines, human and animal interests, welfare, and safety come first. They also cover research involving the use of human biological materials and data such as stem cell research using either living or deceased people's stem cells, as well as research using human embryos, foetuses, foetal tissue, reproductive products, and embryos (Department of Health, 2015). Additionally, the guidelines have a special chapter (Chapter 6) to support RECs and researchers with protocols using qualitative research methods. Furthermore, according to the guidelines, all RECs and ARECs (Animal Research Ethics Committees) that review research involving human participants and animals must be registered with the NHREC (Department of Health, 2015).

The literature indicates five interrelated themes on graduate students and research ethics, namely, teaching approaches, curriculum content, research relationships, learning environments and the REC process. However, research on students’ experiences with the research ethics process is inadequate (Petillion et al., 2017). A discussion of these themes from literature follows.

### Teaching Approaches

Previously, postgraduate students have complained about not receiving or receiving little training on how to conduct responsible research (Vasgrid, 2007). This is changing because compulsory training on research ethics is now provided to postgraduate students (Fisher, Fried, Goodman, & Germano, 2009; Swedan et al., 2020). The teaching of research ethics is done in diverse ways, including guest lectures, lectures, assignments, case studies, workshop, presentation, and online programmes (Naimi, 2007; Petillion et al., 2017; Swedan et al., 2020). There are varying views on the content of research ethics training. Plemmons and Kalichman (2013) found that research ethics instructors had different views on the content to be included in research ethics training programmes. The guidelines provided by Bowater and Wilkinson (2012) indicate a positive and supportive learning environment as the key foundation required for teaching research ethics. Internationally funded research ethics training initiatives are available in South Africa.

For example, Advancing Research Ethics Training in Southern Africa (ARESA) that developed a leadership programme in Bioethics in Southern Africa from 2017–2022 (ARESA, n.d.), and The South African Research Ethics Training Initiative (SARETI), which offers a offers modular, academic and practical learning programme aimed at scientists, health professionals, ethics review committee members, public health staff, social scientists, philosophers, ethicists, health journalists, lawyers, and others who have a direct impact on health (SARETI, 2022).

Additionally, online, certificate-generating research ethics modules based on South African ethical guidelines and regulations such as the ethics training provided by Training and Resources in Research Ethics Evaluation (TRREE, 2021), are also available free of charge to students and academics. The training by TRREE provides basic research ethics training. Some South African RECs make completing this training a basic requirement for all ethics applicants.

### Curriculum Content

A positive relationship between a supervisor and their student is also important. Supervisors provide mentoring to their students through practical guidance such as guiding the student on the application of the research ethics policies of the institution.

Additionally, supervisors also model exemplary responsible conduct of research behaviours (Fisher, Fried, & Feldman, 2009; Fisher, Fried, Goodman, & Germano, 2009). This “hidden curriculum” helps students learn the actual hands-on application of research ethics (Fisher, Fried, & Feldman, 2009; Fisher, Fried, Goodman, & Germano, 2009). However, a study by Jordan and Gray (2012) found that greater levels of training on research ethics received by graduate students lowered the students’ opinions of the ethicality of other senior researchers such as their supervisors.

### Learning Environments

Another crucial element of responsible conduct of research socialisation is the academic department's climate. The ethical research climate is implemented through students’ and academics’ behaviours that respect and comply with research ethics values throughout the academic department (Petillion et al., 2017). The importance of the academic department's climate was highlighted by Anderson et al. (1994). Their results showed that, across all scientific disciplines, the departmental climate was the most critical factor in postgraduates’ dishonesty. Aspects such as the communication of the codes of ethical conduct to students, formal policies on research misconduct, students’ ability to seek help from research ethics committees are made easy. Further, the help students and staff can get from the department in addressing ethical disputes is enabled (Petillion et al., 2017). According to Fisher, Fried and Feldman (2009), students gain a positive attitude towards research and research self-efficacy when departments communicate research ethics adherence and support.

### Research Relationships

According to Van Den Scott (2016), postgraduate students complain about the lack of support they receive from their supervisors on how to interact with the research ethics committees regarding the research ethics application process. Supervisors rarely mentioned or advised students about the research ethics committee. Therefore, students manoeuvred on their own or used the information they gained from research ethics workshops. Additionally, students were often influenced by the research ethics committee decisions when they formulated their research topics. More students steered away from conducting research with vulnerable and protected populations, despite the need for and importance of researching them (Van Den Scott, 2016). A study by Iwu et al. (2019) reported that postgraduate students found the ethics clearance process “demonic”. Petillion et al. (2017) stated that postgraduate students are likely to have a positive experience with the research ethics application process if the research and ethics committees provided the students with research ethics knowledge and negotiated the ethics review process.

## Methodology

### Study Design

This was a qualitative exploratory study to investigate postgraduate students’ experience with research ethics to gain insights into the challenges encountered when applying for ethics clearance and how they overcame them. The study was underpinned by the interpretivist paradigm. This paradigm argues that it is only through subjective interpretations that reality can be fully understood. Researchers using interpretive methods study social reality by applying an open-ended approach to data collection and a flexible research design (Usher, 2020).

### Recruitment of Study Participants

Purposive and snowball sampling of postgraduate students who had completed their postgraduate studies in different academic disciplines, in different higher education institutions in South Africa was employed. Students who graduated between 2018 and 2021 were a targeted sample since they had recent research ethics exposure to reflect on and could provide rich data related to the study's research aim. Participants were recruited using Twitter because postgraduate students who have graduated are less likely to be in contact with their institutions after graduating (Wunnava & Lauze, 2001; Petillion et al., 2017). Therefore, using social media was determined to be an effective way to recruit this population (Guillory et al., 2018; King, O'Rourke & DeLongis, 2014). A Twitter account (@pgResearchEthics) was created specifically to recruit participants. A pamphlet detailing the project and contact information was posted on Twitter account's feed and stories. Hashtags relating to postgraduate studies and research ethics were used to increase the reach of the target population. Additionally, the researchers also posted the pamphlet on their personal accounts. The use of the twitter account to recruit participants enabled not only virtual snowball sampling to be feasible through retweets and tagging but also the expansion of the sample size and the scope of the study and reduce costs and time. To participate in the study the participants had to be 20 years of age and older and graduated with a Masters or Doctoral degree from a South African university or university of technology from 2018 to 2021. The participants studied a range of disciplines as indicated in Table 1.

**Table 1.** Study Disciplines.

Study discipline	Count	Provinces	Qualification
Chemistry	1	Gauteng	PhD
Entrepreneurship	1	Gauteng	PhD
Environmental Sciences	1	Gauteng	PhD
Human Resources Management	1	Limpopo	Masters
Management Studies	1	Gauteng	Masters
Marketing	2	KwaZulu-Natal & Gauteng	PhD & Masters
Population Studies & Development	1	NW	Masters
Public Administration & Management	1	Gauteng	Masters
Strategic Management	1	Gauteng	Masters
Supply Chain Management	1	KZN	Masters
Zoology	1	CT	Masters
Total	12		

Hennink and Kaiser (2022) confirmed that qualitative studies can reach saturation at relatively small sample sizes of 9–17 interviews. Therefore, the sample size was also based on the principle of saturation (where no new information emerges in interviews). Twelve participants were included in the study as saturation was reached. If saturation had not been reached after the twelve planned interviews, the number of participants would have been increased. The participants were from eight different institutions in five provinces. Eight participants were masters graduates and four were doctoral graduates. Participants who expressed an interest in participating were given an informative letter, a consent form, and a convenient interview day and time. The participants presented their certificates at the interviews to confirm their qualifications.

### Data Collection

Semi-structured interviews were conducted on MS Teams for the safety of the participants and researcher from Covid-19. Each interview lasted 50–60 min and was audio-recorded with the participants’ consent. One researcher conducted all the interviews. All responses from the participants were unstructured.

### Data Analysis

The audio recordings of the interviews were transcribed. The researchers started by reading all transcripts to familiarise ourselves with the data, mark, and memo the transcripts. Deductive coding was used. Pre-determined themes were developed from literature. The first set of codes was generated by open line-by-line coding. To identify themes related to participants’ experiences with research ethics, the codebook was updated. In the findings, the verbatim quotes from the participants are used to support the themes. To support the findings, verbatim quotes were used. To confirm the reliability and dependability of the results, member checking was used (Cohen & Crabtree, 2006). A draft of the findings was sent to participants for feedback for the researchers to ascertain whether the respondents’ views aligned with those represented by the researcher. No changes were expressed by the participants.

Ethics approval was received to conduct the study**.** All participants signed an informed consent form before participating in the interviews. They were also informed of the voluntariness of their participation and confidentiality of the information provided. The names of the universities have not been provided for confidentiality reasons. The interview guide explored the students’ experiences with research ethics. Pseudonyms were allocated to each of the participants. The researcher sought to increase validity and trustworthiness (Higson-Smith et al., 2000) by using member checking, which affords participants the opportunity to review their statements for accuracy (Dudovskiy, 2016). Participants were also given the option to go through their transcripts and coding to clarify any misrepresentations. Credibility was also ensured by data source triangulation whereby multiple sources of data were used to validate the conclusions of the findings (Pitney & Parker, 2009). The various sources of data that were included in the research for triangulation were literature, interviews, and documents. To ensure rigour (Strauss & Corbin, 1990), the researcher analysed the information and the data obtained from the first three interviews immediately to facilitate subsequent data collection.

## Findings

Based on the data analysis seven themes emerged. These were research ethics in the curriculum, support received, understanding of university research ethics policy, experience of the ethics application process, perceptions of research ethics, overall experience with research ethics and recommendations from participants.

### Research Ethics in the Curriculum

Most participants (58%) indicated they had not received any research ethics training, with some declaring that the institutions left it to the students to discover, read and understand research ethics principles on their own.

“No modules for masters or lecture with regards to research ethics…Nobody taught me. I had to read and understand (research ethics) on my own” – P7.

“There were no workshops on how to do your research or research ethics. You had to learn on your own. The supervisor assists you with what to write but you must find your own way” – P3.

However, some participants indicated they had attended workshops where they were introduced to research ethics and assessed on their understanding of research ethics principles.

“Part of my proposal module provided supplementary workshops on how to adhere to research ethics and policies at South Africa College of Economic and Management Sciences university and you are also assessed for our understanding on research ethics” – P9.

“I worked at South Africa College of Economic and Management Sciences University before as a graduate assistant and we went to research ethics workshops, and I also worked in the department that dealt with research ethics I was exposed to research ethics and requirements” -P5.

### Support Received from Supervisor

Most (69%) participants reported that they had received general guidance from their supervisors, which included encouragement to comply with research ethics.

“Although not explicit, the whole idea from my supervisor was that I must always do my research ethically” -P8.

In some cases, the supervisor explained how research ethics principles were to be applied during the research project.

“Mainly the supervisor provides advisory capabilities from his side because that's his duty. And to explain to you these things I have just spoken about in terms of ethics. That's how far they can go, I think”. -P1

Thorough guidance on ethics occurred where the supervisor happened to be part of the REC.

“I was fortunate that my supervisor was part of the department's ethics representative, so I had good guidance that was thorough”. -P11

Apart from guidance and helping students understand research ethics, some supervisors also assisted with practical steps in the research ethics clearance process, such as communicating with the relevant bodies on behalf of the students.

“My supervisors guided me and supported me and helped me by sending emails to the institutions I needed to get approval (gatekeeper permission) from”. -P2

Most (58%) of the participants highlighted some gaps in the teaching or support through the research process. The gaps that were identified related to teaching or support with the research ethics process. One of the issues highlighted was inconsistencies in the level to which research ethics was adhered to, even within the same institution.

“There are inconsistencies and different levels of compliance to research ethics depending on the supervisor. Also, different departments used different procedures which results in the inconsistencies”. -P9

“The gaps I have seen is that mentoring, and guidance being lacking…The research was tricky. Data collection, handling participants was challenging because I had no previous experience. After all, you don’t know what you don’t know. The theory on research ethics is different from practice. Step by step guidance would be good”. -P7

There was a sentiment that understanding of research ethics was lacking even among some supervisors and institutions broadly.

“I fear that even the supervisors themselves don’t understand how the ethics go…So, there might be a blind spot there. Institutions themselves need to be taught about research ethics, especially when it comes to intellectual property”. -P1

### Understanding of University Research Ethics Policy

Most (67%) of the participants had not read their institution's research ethics policy. The participants reported they had not read their institution's research ethics policy before commencing the ethics application procedures, with some proclaiming they did not even know if such a policy existed at their institution.

“I didn't think about it (reading the research ethics policy). I don't know if it exists. Supervisors should be the ones to let me know about it”. -P2

“I didn’t because no one told me about that. What I'm saying is that maybe the university itself just produced documents that they don’t even use. Perhaps it's there, I've never seen it”. -P1

Consistent with a lack of awareness and/or lack of direct instructions to read the policy, some of the students took a cursory approach to perusing the documentation they had received as part of the ethics approval process.

“I just received a thick document before my ethics committee approval. I think it was in there”. -P6

“I didn't read the policy, but what happened is that I asked my supervisors which documents I should complete. That was it”. -P12

In the group of participants who had read the research ethics policy before embarking on the ethics clearance application process, one highlighted that their institution made the reading of the policy mandatory before one could seek clearance.

“Yes (I read the research ethics policy). It was a pre-requisite because we got access to the research ethics policy before you apply for ethical clearance. Once you pass then you submit your ethical clearance form, then wait for feedback”. -P9

In one case, the research ethics policy had been read in the process of a general familiarisation with broader institutional policies.

“I actually went through all of South Africa College of Economic and Management Sciences university's policies before registering for my qualification”. -P8

In another case, the student and the supervisor had gone through the policy together.

“Yes, I did (read the research ethics policy). I went through the policy with my supervisor”. -P5

### Application of Research Ethics Principles in the Research Project

Most (72%) participants mentioned having observed one or more of the standard research ethics principles that included obtaining informed consent, ensuring respondent confidentiality and anonymity, non-misrepresentation, and non-plagiarism.

“Referencing and not plagiarising, using consent forms to get data and *confidentiality”. -P3*

“The typical principles I followed were simply that I was to report my results truthfully without ‘cooking’ any outcome”. -P10

One participant regarded the process of seeking and obtaining ethics clearance itself as part of observing research ethics principles in their research project.

“By having mandatory research ethics approval from the committee…Even the ethics committee feedback was just minor corrections”. -P6

Another shortcoming was that the ethics clearance procedure sometimes took too long.

“The ethics clearance process takes long…a whole year and I don’t know why. This needs to be addressed. From the start of your first submission to the REC I waited 6 months and I got feedback and resubmitted then waited another 4 months after this second submission”. -P2

There was, however, some acknowledgment that possibly not all ethics principles were fully applied to their projects.

“I tried to be ethical but I also had a research assistant so I can't say that they were also ethical all the time you know. Because a lot can happen. You give people questionnaires to collect data for you. And people just sit somewhere there as they start completing those questionnaires”. -P12

### Ethics Application Process

The participants shared both positive and negative experiences with the ethics application process. Among the positive experiences was that the participants found the ethics application process to be streamlined and simple, especially when the supervisor led the process.

“At South Africa College of Economic and Management Sciences University the process is streamlined and not as tedious. You work with your supervisor who leads the completion of the form and sits in the ethics committee for feedback and relays the feedback to you (student). Had the supervisor not taken the lead role, feedback from the committee would have come back with lots of amendments if the student submitted”. -P9

There was also reference to the REC providing a requirements checklist, thus making the process clear.

“It was easy because of the system that they used at South Africa College of Economic and Management Sciences university. They give you a checklist and then you also submitted a proposal and then in that proposal you need to have a section that deals with ethics, so they tell you what to do, to cover, and then they also give you the checklist, so it was not that complex”. -P12

The provision of a checklist as above was also said to have aided the quick and efficient granting of ethics clearance.

“It was easy because everything is clear. There's a fact sheet that is provided to students. I got my letter of clearance the first time around”. -P11

However, there were some negative experiences with the ethics application process, which included finding the process complicated and time-consuming.

“I found the application process a bit complicated and time-consuming. It *requires a lot of work to complete and complete successfully”. -P8*

The tediousness of the process was partly attributed to the requirement of “too much” information.

“The application form demanded a lot of work from the student. The quantity of information required makes it difficult to get approval the first time around and we can't shorten the form”. -P8

“It was time-consuming and laborious because the ethics committee took 3–4 months to process and grant approval and this delayed my study”. -P7

### Postgraduate Students’ Perceptions of Research Ethics

Most (93%) participants had positive perceptions of research ethics. Among the positive perceptions of research ethics were that it was important and necessary, it safeguarded institutional integrity, it protected researchers and participants, ensured data integrity, and promoted accountability. Participants said,

“Ethics are very important. As institutions of higher learning, we need to emphasise and amplify issues of research ethics. We need to talk more about issues of research ethics at undergrad because the transition from undergrad to postgrad is hard, for example, having to understand and deal with plagiarism”. -P8

“I think it's good. I think it's a necessity for the operation and safeguarding the institution itself and protecting the student”. -P1

“It needs to happen, although it is a lot of work. They (research ethics) are there to protect participants and the researcher”. -P4

“They are very significant especially because, in this era of the information age, one can just copy and paste information from google and make it as if it is their work. We need research ethics”. -P9

Among the negative perceptions of research ethics were that the ethics clearance procedures are complicated, the process is too strict, it is difficult to adhere to or apply all the ethics principles and there was a lack of adequate training on the ethical clearance application process. The participants stated the following,

“Things have now become very strict though…Things must be strict but also allow for flexibility”. -P6

“It's difficult to get it right. It looks easy in the paper but practically it's more challenging, for example making sure you don’t lead participants in their responses. The university does not properly equip you to handle ethics”. -P7

“The application process can be simplified. Some things are just not necessary…I think they might just simplify the application and focus on what is useful. Or maybe what's most important”. -P12

### Recommendations

Participants proposed the following recommendations that would make the research ethics process a positive experience. The recommendations included the introduction of research ethics at the undergraduate level, inculcation of a culture of research ethics at institutions, provision of more training and support concerning research ethics and the alignment of research ethics to the local context. This is what some participants said,

“Lecturers and supervisors have to convey the importance of research ethics to students all the time…it is an important but an undervalued element of research”. -P11

“We learn of research ethics at postgraduate level. The sooner research ethics are introduced at undergrad the better. I think the dropping out at postgrad is not from the pressure that comes with research but from the lack of support”. - P9

“I think they should be more workshops…You understand what ethics is, but I think the practical side of applying ethics should be emphasised, you know. There should be workshops”. -P12

“Most research guidelines are from a western perspective and in the current discourse of decolonisation, this must also be looked at in terms of research ethics that align to our context”. -P9

## Discussion

The purpose of this study was to explore a sample of South African postgraduate students’ experiences with research ethics. Although some participants in the study indicated they had received training on research ethics, it was concerning that more students had not received any ethics training. This was concerning because most of the participants were masters’ graduates where understanding of research ethics shoud be understood and applied. The study by Charpentier-Jiménez (2023) also reported a lack of ethics training with the postgraduate participants in their study. The lack of ethics training among some participants may be attributed to students’ ignorance of ethics training, as suggested by Kouritzin and Nakagawa (2018) and Hofmann and Holm (2019). The current study's findings are consistent with findings by Janakiram and Gardens (2014) who also noted that ethics training should be included in the early stages of postgraduate studies.

It was expected that the participants would mention their supervisor as a source of support. This finding was similar to that of Davis et al. (2021) who found that support from supervisors was a key influence on postgraduate students’ experience with research ethics. The reason is that the supervisor serves as the primary source of intellectual advice, support, and guidance for the research student and, as such, has a significant impact on training and researcher development. (McEvoy et al., 2018). However, the current study also points to the lack of basic support some students received during their research degrees, as also found by Tobbell and O’Donnell (2013) and Petillion et al. (2017). The negative perceptions of research ethics expressed by the participants in the present study can be easily addressed by institutions in that institutions should consider ways to make the ethics application process easier to complete. This will help expedite ethics approval. Similar to this study's finding, Petillion et al. (2017) also observed that inexperienced researchers require detailed information on the ethics review procedure and the completion of application forms. Wassenaar and Mamotte (2012) and Wassenaar and Slack (2016), point to the need for RECs to enhance and streamline bureaucratic processes, aiming for optimal efficiency to achieve its main goal of safeguarding the safety, dignity, and well-being of research participants. Furthermore, Wassenaar and Slack (2016) propose that undergraduate and postgraduate research training programmes should allocate additional time for research ethics training. They suggest the use of a structured framework to ensure a systematic approach to such training.

Participants in the current study showed a positive attitude towards research ethics, similar to the studies by Davis et al. (2021). However, unlike the study by Kumbhar et al. (2020), where participants suggested that research ethics is extremely important and should be taught as a mandatory postgraduate module, the participants in the current study suggested that research ethics should be introduced at the undergraduate level. This suggestion by the participants shows that students want to be better prepared to deal with research ethics when they reach the postgraduate level.

## Educational Implications

The findings of this study shed light on the gap and students’ needs regarding ethics training programmes in South Africa. Such efforts could improve students’ awareness of the role and importance of research ethics when undertaking their research projects, thus ensuring ethical research is conducted. All institutions should adopt the use of the TRREE training and make it compulsory for postgraduate students to undertake this training as it is freely available online and can be concluded at one's own pace.

## Best Practice

The inclusion of a compulsory research ethics course or module that all postgraduate students need to attend could go a long way in ensuring that all students undertaking research have gone through and understand the importance of conducting ethical research. The proof of attendance of the compulsory course/module could also be included as a requirement when applying for ethics clearance. The training provided to students should also focus on the practical application of ethics to help students understand how they can deal ethical challenges while in the field and after data collection. Training also needs to be provided to supervisors so that they can provide adequate support to students on how to conduct ethical research. We are of the view that institutions also need to provide standard training and guidelines to RECs to enable the consistent adjudication of ethics clearance applications to ensure the consistent application of the institutions’ rules and guidelines.

## Research Agenda

Future studies involving students from more institutions and disciplines are recommended. This could involve a quantitative study as generalisations to other South African universities not included in the current study can be made owing to the larger sample size.

## Limitations

This study had limitations. A small number of participants was sampled. Although the sample included participants from different universities and disciplines, it may not indicate the experiences of all the postgraduate students in other institutions and disciplines, thus limiting the generalisability of the results. The use of snowball sampling could be the reason why most participants may have invited participants from their own disciplines. This could explain the reason why Table 1 is dominated by participants from business disciplines and very few from the health and humanities disciplines. This could also explain why most participants were not familiar with research ethics especially if their research projects did not include interaction with humans.

## Conclusion

The topic reported in this paper reflects the importance of conducting ethical research. Published research findings on South African postgraduate students are limited. We found that postgraduate students generally agreed that research ethics was important, but they expressed a need for more support and guidance on research ethics in the form of more training, especially on the practical application of research ethics. The participants also needed simplified information required on ethics application forms and the ethics review process as this would reduce delays in obtaining ethical clearance. Furthermore, recommendations for institutions were presented. These included the introduction of research ethics at the undergraduate level, the development of an institutional culture of research ethics, the provision of greater training and support regarding research ethics, and the adaptation of research ethics to the local situation. Therefore, the findings in this study hopefully contribute to a better understanding of postgraduate students’ experiences with research ethics in the South African context. The findings in this study may be beneficial to institutions seeking to better understand postgraduate students’ experiences of the research ethics process.
